# Non-local attention enhanced deep learning for robust cyberattack detection in industrial IoT-based SCADA systems

**DOI:** 10.1038/s41598-026-37146-1

**Published:** 2026-02-09

**Authors:** Mustafa Tahsin Yilmaz, Onur Polat, Enes Algul, Ferdi Doğan

**Affiliations:** 1 Department of Industrial Engineering, Faculty of Engineering, King Abdulaziz University, Jeddah, Saudi Arabia; 2 Center of Research Excellence in Artificial Intelligence and Data Science (AIADS), King Abdulaziz University, Jeddah, Saudi Arabia; 3 Department of Computer Engineering, Bingöl University, Bingöl, 12000 Turkey; 4 Department of Computer Engineering, Adiyaman University, Adiyaman, 02040 Turkey

**Keywords:** Intrusion detection system, Industrial internet of things, Supervisory control and data acquisition, Deep learning, Non-Local attention, Energy science and technology, Engineering, Mathematics and computing

## Abstract

Industrial Internet of Things (IIoT)-enabled Supervisory Control and Data Acquisition (SCADA) systems are pivotal for real-time monitoring and control in critical sectors like energy, manufacturing, and water management. However, their connectivity and complexity expose them to cyber threats, including zero-day vulnerabilities and advanced persistent threats (APTs). Traditional security measures, like signature-based intrusion detection systems (IDSs), are inadequate against dynamic attacks. This study introduces DeepNonLocalNN, a deep learning model combining convolutional neural networks (CNNs) with non-local attention blocks to capture local patterns and global dependencies in IIoT network traffic. Evaluated on the WUSTL-IIoT-2021 dataset, DeepNonLocalNN achieved strong performance, with an accuracy of 0.9999, a receiver operating characteristic-area under the curve (ROC-AUC) of 1.0000, and a macro F1-score of 0.93, outperforming baseline models such as NonLocalNN, CNNWithAttention, ResidualAttentionNetwork, and Long Short-Term Memory (LSTM). Notably, it excelled in detecting minority attack classes, including Backdoor (F1: 0.73) and Command Injection (CommInj, F1: 0.92), addressing class imbalance. The model’s scalable architecture, leveraging non-local attention and regularization, provides a high-performance solution for SCADA security in IIoT environments. Future work will focus on adapting the DeepNonLocalNN approach to real-time intrusion detection. It also aims to reduce the computational cost for resource-constrained PLCs and RTUs in SCADA systems. We also aim to validate this model on various industrial datasets and SCADA environments.

## Introduction

### Industrial IoT and SCADA systems

**Fig. 1:**
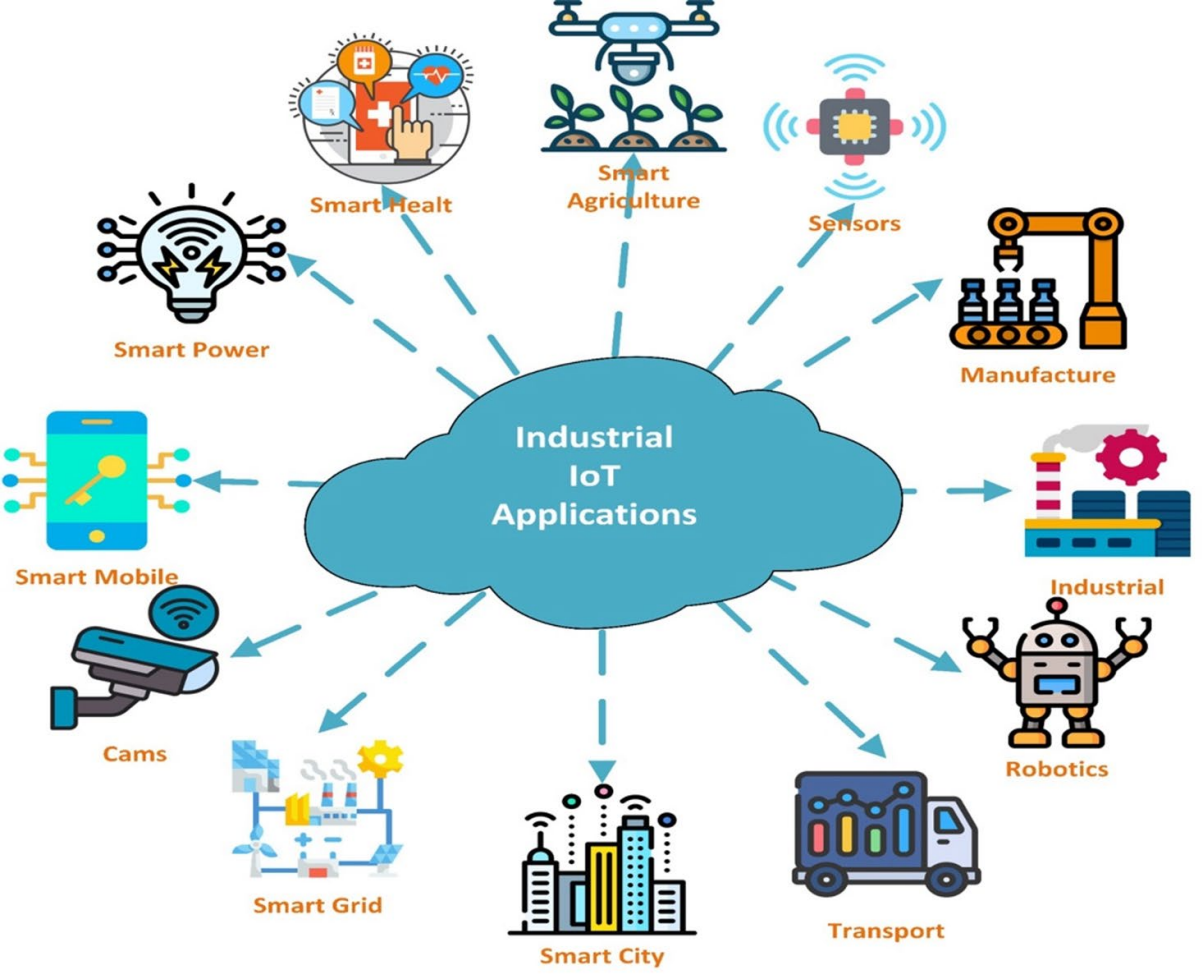
Industrial IoT devices and their application areas.

The Internet of Things (IoT) is a virtual environment where devices connected to the internet environment are located that enable devices to communicate with each other. There are many devices within these technologies. These are sensors, smart devices, cameras, and many devices connected to the internet used in homes and workplaces. They are used in many areas such as smart homes, the healthcare sector, systems brought by Industry 4.0, smart cities, and agriculture. IoT devices are part of real-time monitoring and control processes that are based on efficiency and provide cost savings^1^. Figure 1 shows the applications of industrial IoT devices. IoT devices are devices used in daily life. Industrial IoT devices, on the other hand, are devices located in industrial environments that enable data to be sent to a central hub. IIoT devices are used in industrial workspaces such as energy, transportation, shipping, and distribution. The hub where this data is collected and sent is called a SCADA system. It is responsible for processes such as data acquisition, network traffic control, and monitoring of connected devices.

Industrial automation has a centralized control structure. These automations are integrated systems created to provide central or remote monitoring and control of large-scale enterprises are called SCADA systems. SCADA systems carry out the monitoring and control processes of devices in the field. Such systems include PLC, sensors, RTU, servers, data storage systems, firewall, HMI (Human Machine Interface)^2^.

SCADA systems contain many devices and management tools. Especially IoT devices are the basic parts of SCADA systems. Data is received or sent from many IoT devices in a SCADA system. Data is analyzed and commands are sent with the central control system. IoT devices operate according to the commands received. The central control system monitors and controls these systems from their internal network or by providing remote connections. The fact that SCADA systems allow remote connections increases the transition of industrial production systems to these systems. It can be used in many areas such as energy transmission and distribution, factories, chemical plants, water treatment and distribution facilities, transportation and control systems, smart building systems, agricultural irrigation and greenhouse management.

### Cybersecurity challenges in IoT-Enabled SCADA

Having such a widespread usage network and the possibility of remote access require such systems to be strong in terms of cyber security. An attack on the system will target the central management connected to the system or the IoT devices in the system. The increasing number of cyber attacks worldwide shows that such systems are at risk. SCADA systems where IoT devices are active require both the central management and the connected IoT devices to be strong in terms of security. Especially real-time systems where IoT devices are located are more attractive to attackers. The fact that the hardware software of IoT devices is not up to date, weak authentication processes, and attempts to integrate old-type devices into the new system bring security vulnerabilities. In addition, the low processing power of some IoT devices does not support some security measures. This situation in the network can make the entire system vulnerable. The entire system can be taken over by attackers finding a gap in the system and entering the system. The production line can be stopped, systems can be operated incorrectly, and IoT devices can exhibit approaches that can damage the infrastructure. This increases the importance of security in SCADA systems where IoT devices are located^3^.

### Limitations of traditional security mechanisms

Classical security measures are built on signature-based detection systems, firewalls, and static access control systems. These methods are old-style security systems and are effective in classic attack types. However, they are insufficient against dynamic and complex attacks involving industrial IoT devices. Classical security systems operate with a rule-based management. However, today’s cyber attacks, zero-day vulnerabilities, phishing, and advanced persistent threats cannot be detected according to these rules. They need to be analyzed. However, it is not possible to monitor the analyses manually. Such advanced cyber attacks can be detected with machine learning and artificial intelligence systems. Machine learning and artificial intelligence-based systems offer an autonomous security approach. Real-time monitoring of cyber attacks in SCADA systems where IoT devices are located, anomaly detection, zero-day threats, and phishing threats can provide warnings. Artificial intelligence techniques that learn from past data also allow the detection of unknown threats. Thus, it creates a detection and defense system against known and unpredictable attacks^4^.

### Role of artificial intelligence in intrusion detection

In IoT-based SCADA systems, artificial intelligence-based detection systems provide an important solution for intrusion detection and attack mitigation strategies. It has advanced detection capabilities. It analyzes network traffic and provides high-precision detection methods against threats. Many artificial intelligence methods such as XGBoost^5^, random forest algorithms^6^, Bi-LSTM^7^, ensemble learning^8^ can be used. It is successful in detecting complex cyber attacks. It can learn new types of attacks. It can also provide a comprehensive security environment against different types of attacks^9^.

### The aim and contributions of the study

The main objective of this study is to develop an intrusion detection system for IIoT-based SCADA systems, considering the fundamental problems in network traffic. A model capable of identifying both common and rare attacks, despite the complexity of network traffic, is proposed. The contributions of this study are as follows:

#### A new hybrid architecture

The DeepNonLocalNN model with non-local attention blocks that can learn local and general features is presented.

#### Using a realistic dataset

The proposed model has been tested with the WUSTL-IIoT-2021 dataset containing network traffic of SCADA-based IoT devices and has demonstrated its validity by achieving high performance.

#### Comparative analysis with other models

It has been compared with LSTM, CNNWithAttention, ResidualAttentionNetwork models frequently used in the literature. F1-score, ROC-AUC, accuracy metrics have been measured and the proposed model has shown better performance than other models.

#### High performance in classes with small sample numbers

High performance was demonstrated in Backdoor and CommInj attack types with small sample numbers, focusing on the class imbalance problem.

#### A scalable architecture

The design of the model is designed to work with large amounts of data. It is also suitable for heterogeneous IoT data. Thus, it facilitates integration into real industrial applications.

The remaining sections of this paper are organized as follows. Section Related works examines relevant literature studies, attacks on SCADA systems, machine learning and deep learning-based intrusion detection approaches, and the IIoT-SCADA datasets used in such studies. Section Materials and methods provides a detailed description of the WUSTL-IIoT-2021 dataset, preprocessing processes, the underlying models used, the proposed DeepNonLocalNN architecture, and the experimental setup, along with comparative results. Section Results and discussion provides a discussion and comparative analysis of experimental results. Section Conclusion presents the conclusions of the study, and Sect. Limitations and future work discusses limitations and future work.

## Related works

There are various attacks against SCADA systems in the literature. Some of these attacks are reconnaissance attacks, response and measurement injection, command injection, denial of service (DoS), man-in-the-middle attacks (MITM), replay and fragmentation attacks, cyber-physical attacks.

Reconnaissance attacks are carried out to collect information to detect vulnerabilities in the network environment. Different network scanning tools are used to obtain information about the system architecture and to find open connections^10^. Input and measurement injection injects data to obtain and manipulate system information. These types of attacks cause the decisions to be taken in the system to be wrong. It can cause physical damage or system outages^11^. In command injection attacks, the aim is to change the processes in the system with unauthorized commands^12^. DoS and DDoS attacks are attacks that occupy network traffic and cause the system to stop^13^. MITM attacks aim to change the data of the components in SCADA systems^14^. Covert attacks such as Hijacking and Blackout change the parameters by taking advantage of network protocol weaknesses and stop the detection of processes^15^. Replay attacks allow data packets on the network to be retransmitted after being captured. Data packets are corrupted, causing a system crash^16^. Cyber-physical attacks target physical components to change the properties of IoT devices to gain unauthorized access^17^.

The healthy operation of IoT-based SCADA systems depends on many factors within the system. Field end devices, communication protocols, servers and control centers, cloud structures and remote access points, and data storage points are targeted. The presence of many connected factors has made SCADA systems a target for attacks^18^.

### Attacks on SCADA systems

Many attacks carried out through malware can cause serious damage to such systems^19^. The cyber attack carried out in Ukraine in 2015 was carried out through malware. Biswas, The security vulnerabilities of the technological devices targeted in the attack were revealed in a study^20^. Camargo at all. stated that the malware disrupted the Modbus-TCP communication. This situation can cause delays and interruptions, and the system is affected^21^. Phishing attacks are accessed by taking advantage of human-based vulnerabilities. Ali stated in his study that these attacks are based on deceiving people in various ways. He emphasized that this situation makes phishing detection quite difficult^18^. In order to prevent such attacks, Sen at all. offered solutions such as multi-factor authentication, employee training, and e-mail filtering^22^. Srivastava at all. stated that DDoS attacks cause service interruptions by targeting IoT devices in the network in particular. It can create a bottleneck in the network and try to render the network dysfunctional^23^. Attacks on SCADA systems, targets in the system, methods used and their effects are given in Table 1.

**Table 1:** Representative cyberattack types targeting SCADA systems and their Impacts.

Attacks	Target/Method	Impact
Black Energy Attack^24^	Ukraine power grid using malware.	Infected over 200,000 people. Caused power outages.
Crash Invalidation Attack^25^	Ukraine power grid station to cause intermittent power outages	Demonstrated the highly destructive potential of attacks on infrastructure.
Modbus TCP Penetration^26^	Tested Modbus/TCP protocol vulnerabilities using test tools to penetrate SCADA systems.	Compromised system integrity and availability. Identified vulnerabilities.
Wastewater Treatment Plant^27^	Detected a Denial of Service attack using live memory dump analysis.	Water treatment was disrupted. Demonstrated the need for forensic investigation in ICS environments.
False Data Injection^28^	Injected false data into a photovoltaic (PV) production meter for grid estimation.	Settings began to malfunction. Lead to cascading failures and voltage sags.
IEC 60870-5-104 Attack^29^	Exploited vulnerabilities in IEC 60870-5-104 SCADA protocol	Demonstrated the feasibility of attacking smart grid substations using Hardware-in-the-Loop (HIL).
Modbus Buffer Overflow^30^	Exploited a buffer overflow vulnerability in Modbus protocol communications.	Disrupted service and caused potential system crashes.
Denial of Service^31^	Flood and resource exhaustion attacks on energy meters using Modbus TCP	Caused communication disruption and demanded computing resources.
Man-on-the-Side^32^	Injected fake command responses into an industrial control network.	Allowed attackers to manipulate system operations without detection.

Some examples of attacks on SCADA systems are given. There are various attacks in these examples. The attacks and their effects were examined one by one and Table 1 was obtained.

### Machine learning and deep learning based detection approaches in IoT-based SCADA systems

The effects of attacks on SCADA systems are increasing. Preventing and detecting these attacks is critical to eliminate possible damage. In recent years, machine learning and artificial intelligence techniques have been particularly successful in detecting attacks. Artificial intelligence techniques provide better results than machine learning techniques. For this reason, the measures taken are often prevented by the success of artificial intelligence models. Artificial intelligence approaches that will be integrated into the system prevent such attacks. Examples of studies conducted with machine learning and artificial intelligence methods have been compiled and turned into a table. These examples are detailed in Table 2.

The table, which includes traditional machine learning methods, deep learning methods, ensemble learning methods and hybrid methods, provides important information. The effectiveness and performance information of each machine learning method in SCADA systems is given. Which methods should be used according to the type of problem is clearly given in this table.

**Table 2:** Comparative table of the use of machine learning and artificial intelligence methods in IoT-based SCADA systems.

Machine Learning Method	Description	Performance
Support Vector Machine^33^	A traditional ML algorithm for nonlinear classification tasks.	High accuracy with feature selection techniques
Random Forest^34^	An ensemble method for processing imbalanced datasets.	High detection accuracy and robustness
Decision Trees^35^	A simple and interpretable classifier for real-time applications.	Improved performance with feature selection
K-Nearest Neighbors^36^	A simple classifier for anomaly detection.	Less effective for low precision attacks
Naïve Bayes^37^	A probabilistic classifier for anomaly detection.	Poor performance in complex environments
Convolutional Neural Networks^38,39^	A deep learning model for feature extraction.	Superior performance with XGBoost
Long Short-Term Memory^40^	Effective for detecting correlated attacks.	High detection rates for real-time applications
Recurrent Neural Networks^41^	Suitable for sequential data analysis.	Effective with ensemble learning methods
Stacked Autoencoders^39^	A deep learning model for unsupervised anomaly detection.	Effective for high-dimensional datasets
Deep Belief Networks^39^	Models complex patterns in network traffic.	Effective with ensemble learning methods
Ensemble Learning^42^	Combines predictions from multiple models for improved accuracy.	High detection accuracy and robustness
Hybrid Models^43^	Combines deep learning and traditional ML techniques.	Improved detection performance for correlated and uncorrelated attacks
Pyramidal Recurrent Units^8^	A deep learning approach for processing irrelevant features.	High detection rates in SCADA systems

Innovative studies in recent years have presented various deep learning-based approaches for security in IoT/SCADA systems. One study indicates that leveraging advanced attention-based mechanisms in industrial systems improves the detection of complex cyberattacks^44^. Another study proposes a hybrid neural network architecture to provide a more robust system in unbalanced attack scenarios. These studies reveal trends toward architectures that reveal features representing local and global contextual relationships^45^. This study appears to align with recent studies.

### Datasets used to detect attacks on IoT-Based SCADA systems

Along with the methods used, the datasets used in these studies are also of great importance. The data to be trained on these methods must be robust and real-world data. The datasets are expected to be correctly classified datasets representing attacks that will occur on the network. The datasets and their features used in the literature to detect attacks on IoT-based SCADA systems are given in Table 3.

**Table 3:** Overview of public datasets used for intrusion detection in IoT-based SCADA systems.

Datasets	Key Features	Evaluation Results
Ton-IoT^45^	Heterogeneous IoT data, DDoS, botnet attacks	99.9% accuracy with DenseNet
UNSW-NB15^46^	Network traffic logs, DDoS, R2L, U2R attacks	98.4% accuracy with Start Time
BOT-IoT^47^	IoT network traffic, botnet attacks	Validated for network forensics
WUSTL-IIoT-2018^5,48^	ICS-specific attacks, SCADA security	99.99% accuracy with Random Forest
CICIDS2017^49^	DDoS, brute force, ransomware attacks	High accuracy with Decision Trees
X-IIoTID^50^	Industrial IoT, multi-stage attacks	Comprehensive for OT-IDS investigation
ICS-SCADA^48^	ICS-specific traffic, vulnerability detection	High accuracy for vulnerability detection
CicDDoS2019^8^	Modern DDoS attacks	High accuracy with deep learning
Gas Pipeline Dataset^51^	SCADA-specific traffic, energy sector attacks	High accuracy with Bi-LSTM and Bi-GRU

Dataset selection is important for evaluating attack detections. Determining the type of attack depends on the characteristics of the research, such as protocols and system architecture. Datasets such as Ton-IoT and UNSW-NB15 are suitable for general IoT-based research. WUSTL-IIoT-2018 and ICS-SCADA are datasets adapted for industrial SCADA systems. BOT-IoT and Gas Pipline datasets are datasets that can be used for attacks specific to botnets and SCADA systems. In the literature studies, it is stated that appropriate datasets should be preferred for attack detection in IoT-based SCADA systems.

### Research gaps

A review of the literature revealed several gaps. The main research gaps in the field of AI-assisted intrusion detection for IIoT-SCADA systems are as follows:

- In literature studies, synthetic data has mostly been studied instead of real world data. Using data from real industrial environments is very important in terms of attack detection.
- Attacks are dynamic with developing technological approaches. Therefore, in many studies, the ability to adapt to dynamic attacks is weak. The dynamic artificial intelligence models to be developed will ensure that the system is kept up to date. The system will be more secure against new attacks.
- Zero-day attacks are weak against existing systems. Studies on detecting abnormalities with artificial intelligence-based approaches should be investigated further.
- Artificial intelligence-based attack detection systems require more resources. Systems expected to be better in terms of hardware may require additional costs to integrate into existing systems. For this reason, new artificial intelligence methods to be developed should focus on models that require less resources.
- There are many different types of attacks on such systems. Combined hybrid approaches should be investigated further for the detection of these attacks. Thus, security can be increased against different types of attacks.

## Materials and methods

### Dataset description

The main reason for selecting the WUSTL-IIoT-2021 dataset used in the experimental study is that it was created in a testbed environment that accurately replicates real industrial systems. Another reason for choosing this dataset is that it was collected under approximately 53 h of operational traffic and real attack scenarios. The dataset underwent pre-processing to remove outliers and corrupted records. This ensured a high-quality and reliable experimental environment. Furthermore, this dataset includes behaviour-based attack categories that remain relevant in IIoT networks today, such as denial of service, scanning, malicious control, and operational manipulation. It therefore reflects the fundamental attack dynamics representing contemporary threats. A review of recent literature indicates that the WUSTL-IIoT-2021 dataset is still widely used as a benchmark dataset due to its realistic industrial network characteristics and reproducibility advantage. In line with the stated reasons, the technical content and data structure of the data set used in the study are summarised below.

The WUSTL-IIoT-2021 dataset^48^, a comprehensive collection of network traffic data from heterogeneous IoT devices specifically designed for IoT security research. This dataset, stored in a single CSV file, contains features such as source and destination IP addresses, timestamps ($\:\mathrm{StartTime}$, $\:\mathrm{LastTime}$), and traffic categories labeled under the $\:\mathrm{Traffic}$ column. The target variable, $\:\mathrm{Traffic}$, represents categorical labels (‘Backdoor’ ‘CommInj’ ‘DoS’ ‘Reconn’ ‘normal’), with the number of unique categories determined dynamically during preprocessing. The dataset’s severe class imbalance and high dimensionality necessitate robust preprocessing and modeling techniques to achieve effective intrusion detection^52^ .

### Basic statistic of the dataset

The WUSTL-IIoT-2021 dataset, utilized in this study, comprises $\:\mathrm{1,194,464}$ network traffic samples from heterogeneous IoT devices, designed specifically for evaluating intrusion detection systems in industrial IoT environments. The dataset includes 44 features, capturing attributes such as source and destination ports (Sport, Dport), packet counts (SrcPkts, DstPkts, TotPkts), byte counts (SrcBytes, DstBytes, TotBytes), network load (SrcLoad, DstLoad, Load), and jitter (SrcJitter, DstJitter), among others. The target variable, Traffic, categorizes network flows into five classes: Backdoor, CommInj, DoS, Reconn, and normal, with a highly imbalanced distribution dominated by normal traffic ($\:\mathrm{221,490}$ samples) compared to attack classes (e.g., Backdoor: 42 samples, CommInj: 52 samples).

**Table 4:** Descriptive statistics of key numerical features in the WUSTL-IIoT-2021 dataset.

Feature	Count	Mean	Std Dev	Min	Skewness	Kurtosis
Mean	1,194,464	1.29E-01	6.86E-01	0.00000	5.432741	2.85E + 01
Sport	1,194,464	5.45E + 04	1.20E + 04	0.00000	6.842448	2.18E + 03
Dport	1,194,464	7.91E + 02	3.30E + 03	0.00000	13.643104	2.06E + 02
SrcPkts	1,194,464	1.67E + 02	5.27E + 04	0.00000	480.812992	2.41E + 05
DstPkts	1,194,464	1.69E + 01	1.14E + 03	0.00000	179.01987	3.49E + 04
TotPkts	1,194,464	1.76E + 02	5.27E + 04	0.00000	480.804408	2.41E + 05
DstBytes	1,194,464	7.60E + 03	7.51E + 05	0.00000	108.298307	1.17E + 04
SrcBytes	1,194,464	1.94E + 04	4.73E + 06	0.00000	347.443668	1.35E + 05
TotBytes	1,194,464	2.78E + 05	1.92E + 07	0.00000	81.039053	7.12E + 03
SrcLoad	1,194,464	1.57E + 07	8.34E + 07	0.00000	5.462176	2.85E + 01
DstLoad	1,194,464	2.22E + 05	7.92E + 06	0.00000	59.669404	3.76E + 03
Load	1,194,464	1.59E + 07	8.37E + 07	0.00000	5.43563	2.82E + 01
SrcRate	1,194,464	3.11E + 04	1.66E + 05	0.00000	5.477397	2.86E + 01
DstRate	1,194,464	4.14E + 02	1.53E + 04	0.00000	61.118732	3.89E + 03
Rate	1,194,464	3.15E + 04	1.67E + 05	0.00000	5.452961	2.84E + 01
SrcLoss	1,194,464	2.29E + 00	2.64E + 01	0.00000	78.372061	6.16E + 03
DstLoss	1,194,464	2.57E + 00	5.26E + 01	0.00000	80.063825	6.97E + 03
Loss	1,194,464	7.17E + 00	2.53E + 03	0.00000	1091.675366	1.19E + 06
pLoss	1,194,464	2.01E + 01	8.08E + 00	0.00000	1.953053	7.40E + 00
SrcJitter	1,194,464	4.70E + 02	4.69E + 02	0.00000	11.935704	4.91E + 02
DstJitter	1,194,464	1.39E + 01	1.49E + 02	0.00000	124.157019	3.23E + 04
SIntPkt	1,194,464	8.25E + 01	5.59E + 02	0.00000	9.798689	1.14E + 02
DIntPkt	1,194,464	8.30E + 00	7.08E + 01	0.00000	61.282852	4.46E + 03
Proto	1,194,464	9.48E + 01	1.62E + 03	0.00000	21.31291	4.57E + 02
Dur	1,194,464	8.56E-01	4.95E + 02	0.00000	792.159465	6.37E + 05
TcpRtt	1,194,464	1.83E-03	5.49E-02	0.00000	52.361414	2.74E + 03
IdleTime	1,194,464	1.55E + 09	2.91E + 07	0.00000	-53.110966	2.82E + 03
Sum	1,194,464	1.99E-01	7.97E-01	0.00000	5.334822	2.73E + 01
Min	1,194,464	1.99E-01	7.97E-01	0.00000	5.334822	2.73E + 01
Max	1,194,464	1.99E-01	7.97E-01	0.00000	5.334822	2.73E + 01
sDSb	1,194,464	1.88E-03	1.85E-01	0.00000	220.346137	5.61E + 04
sTtl	1,194,464	1.29E + 02	2.46E + 01	0.00000	-0.364236	1.62E + 01
dTtl	1,194,464	5.83E + 01	1.86E + 01	0.00000	-2.666484	6.06E + 00
sIpId	1,194,464	3.17E + 04	1.88E + 04	0.00000	0.082482	-1.16E + 00
dIpId	1,194,464	2.96E + 04	2.03E + 04	0.00000	0.07806	-1.24E + 00
SAppBytes	1,194,464	2.19E + 02	2.85E + 03	0.00000	20.196282	4.28E + 02
DAppBytes	1,194,464	7.05E + 03	7.45E + 05	0.00000	108.304287	1.17E + 04
TotAppByte	1,194,464	6.58E + 05	4.17E + 07	0.00000	77.205256	6.53E + 03
SynAck	1,194,464	1.80E-03	5.49E-02	0.00000	52.363086	2.74E + 03
RunTime	1,194,464	1.99E-01	7.97E-01	0.00000	5.334822	2.73E + 01
sTos	1,194,464	7.53E-03	7.44E-01	0.00000	220.741247	5.62E + 04
SrcJitAct	1,194,464	6.19E + 01	4.14E + 02	0.00000	8.148149	7.11E + 01
DstJitAct	1,194,464	2.65E-01	5.00E + 00	0.00000	34.993076	2.65E + 03
Target	1,194,464	7.28E-02	2.60E-01	0.00000	3.287179	8.81E + 00

Table 4 summarizes key statistical properties of selected numerical features, including count, mean, standard deviation, minimum, skewness, and kurtosis. The dataset exhibits significant variability, with features like TotBytes (mean: $\:2.78\times\:{10}^{5}$, std: $\:1.92\times\:{10}^{7}$) and Load (mean: $\:1.59\times\:{10}^{7}$, std: $\:8.37\times\:{10}^{7}$) showing high standard deviations, indicative of diverse traffic patterns. Skewness values, such as $\:480.81$ for TotPkts and $\:347.44$ for SrcBytes, highlight right-skewed distributions, reflecting the presence of extreme values in attack scenarios. High kurtosis, notably $\:1.19\times\:{10}^{6}$ for Loss and $\:6.37\times\:{10}^{5}$ for Dur, suggests heavy-tailed distributions, emphasizing the dataset’s complexity and the need for robust preprocessing to handle outliers and class imbalance.

### Data preprocessing

Data preprocessing was performed to ensure the dataset was suitable for training a deep learning model. The preprocessing pipeline included the following steps:

1. *Missing Value Imputation*: Missing values, which were sparse (< 1% of entries after initial cleaning) and primarily occurred in numerical traffic metrics (e.g., packet/byte counts, jitter), were imputed with zeros. This choice is semantically appropriate because a zero naturally represents the absence of activity in network flows. Alternative approaches such as mean/median imputation were considered but avoided as they could distort the heavily skewed distributions of several features (e.g., TotBytes, SrcBytes). Zero-imputation preserves the original traffic pattern interpretability, which is critical for intrusion detection tasks^53^.
2. *Target Label Encoding*: The categorical target variable ($\:\mathrm{Traffic}$) was encoded into numerical labels using a LabelEncoder, transforming each class $\:\left({c}_{k}\right)$ into a unique integer $\:({y}_{i}\in\:\{\mathrm{0,1},\dots\:,K-1\left\}\right)$, where $\:K$ is the number of unique classes^54^.
3. *Feature Encoding*: Non-numeric features were processed as follows: Timestamp columns ($\:\mathrm{StartTime}$, $\:\mathrm{LastTime}$) were converted to Unix timestamps (seconds since epoch) to obtain numerical representations ($\:{t}_{\mathrm{Unix}}$ $\:=\:\lfloor\:(t-{T}_{epoch}$)$\:/{10}^{9}\rfloor\:$). The $\:\mathrm{Traffic}$ column, if containing a small number of categories $\:\left((\le\:10)\right)$, was one-hot encoded, resulting in binary feature vectors for each category. Other categorical columns (e.g., $\:\mathrm{SrcAddr}$, $\:DstAddr$) were label-encoded to map categorical values to integers^55^.
4. *Feature Normalization*: All numerical features were standardized using the StandardScaler to achieve zero mean and unit variance^53^. The preprocessed dataset was split into training, testing, and validation sets using a 60:20:20 ratio, with stratification to preserve the class distribution.
5. *Data Integrity Verification*:

1 $$\:{I}_{\mathrm{data}}={\sum\:}_{i=1}^{n}\left[\delta\:\left({x}_{i}\right)+\eta\:\left({x}_{i}\right)+\tau\:\left({x}_{i}\right)\right]$$

where $\:\delta\:\left({x}_{i}\right)$ validates data type consistency, $\:\eta\:\left({x}_{i}\right)$ checks for null values, and $\:\tau\:\left({x}_{i}\right)$ verifies structural integrity for each data point $\:{x}_{i}$.

### Deep learning model architectures for tabular data classification

The WUSTL-IIoT-2021 dataset was specifically chosen for this study because it provides a comprehensive and realistic testbed for industrial network security. It features heterogeneous IoT devices and real-world network traffic from a SCADA environment, including challenging conditions such as high data dimensionality and a severe class imbalance. This makes it a robust proxy for evaluating models designed for industrial IoT-SCADA systems, enabling the validation of our model’s ability to handle complex, subtle, and imbalanced attack scenarios.

#### Baseline models

The classification of IoT network traffic, as exemplified by the WUSTL-IIoT-2021 dataset, requires models capable of capturing both temporal dependencies and complex patterns in tabular network flow data. To evaluate the effectiveness of our proposed DeepNonLocalNN model, we selected four baseline models including LSTM, NonLocalNN, CNNWithAttention, and ResidualAttentionNetwork. Each chosen for their distinct architectural strengths in addressing key aspects of IoT traffic classification, such as sequential modeling, global context, local feature extraction, and deep hierarchical processing. These models provide a comprehensive comparison to assess the contribution of non-local attention mechanisms in our proposed approach.

*LSTM*: The Long Short-Term Memory model addresses the temporal nature of network traffic by modeling sequential dependencies across time-ordered observations. This approach is particularly relevant for IoT environments where attack patterns may evolve over time. The model employs a 3-layer LSTM architecture with hidden_dim = 128 units per layer. Each LSTM layer includes forget gates, input gates, and output gates that regulate information flow: $\:{f}_{t}=\sigma\:\left({W}_{f}*\left[{h}_{t-1},{x}_{t}\right]+{b}_{f}\right),{i}_{t}=\sigma\:\left({W}_{i}*\left[{h}_{t-1},{x}_{t}\right]+{b}_{i}\right),{o}_{t}=\sigma\:\left({W}_{o}*\left[{h}_{t-1},{x}_{t}\right]+{b}_{o}\right)$, where $\:\sigma\:$ represents the sigmoid activation. Input sequences of length 10 are generated using sliding window preprocessing, where each sequence represents consecutive network flow observations. The model processes these sequences to predict traffic classification for the subsequent observation, enabling proactive threat detection^55^. The LSTM’s cell state $\:{C}_{t}={f}_{t}*{C}_{t-1}+{i}_{t}*tanh\left({W}_{C}*\left[{h}_{t-1},{x}_{t}\right]+{b}_{C}\right)$ provides long-term memory capabilities, crucial for detecting attack patterns that span multiple time steps. The final hidden state from the last LSTM layer feeds into a fully connected classification layer.

##### NonLocalNN

NonLocalNN serves as a simplified baseline to evaluate the contribution of non-local attention mechanisms without the complexity of deep hierarchical processing^56^. This model isolates the effect of global attention mechanisms in network traffic classification. The model contains a single 1D convolutional layer (1 → 64 channels, kernel_size = 3) followed by batch normalization and one non-local attention block. This minimalist design allows for direct assessment of non-local attention benefits without confounding effects from deep architectures. The single non-local block operates identically to those in DeepNonLocalNN but processes less abstract features. The attention mechanism computes relationships across the 64-dimensional feature space, providing global context for classification decisions.

*CNNWithAttention*: This hybrid architecture combines the local feature extraction capabilities of convolutional neural networks with the global modeling power of multi-head attention mechanisms, representing a popular approach in modern deep learning. A single 1D convolutional layer (1 → 64 channels, kernel_size = 3) with batch normalization extracts local traffic patterns. The moderate channel depth balances representational capacity with computational efficiency. The 8-head attention mechanism (embed_dim = 64, num_heads = 8) enables the model to attend to different representation subspaces simultaneously^57^. Each attention head computes: $\:{\mathrm{Attention}}_{i}=softmax\left({Q}_{i}{K}_{i}^{T}/\sqrt{{d}_{k}}\right){V}_{i},$ where $\:{d}_{k}$ = 64/8 = 8 is the key dimension per head. The multi-head attention processes the permuted convolutional features (sequence_length, batch_size, channels) and returns attention-weighted representations. Global mean pooling aggregates these representations for final classification through a two-layer head (64 → 32 → num_classes).

*ResidualAttentionNetwork*: The most sophisticated baseline model, ResidualAttentionNetwork combines residual connections with attention mechanisms to provide a robust architecture capable of learning complex traffic patterns while maintaining gradient flow through deep networks. The model employs residual connections around the attention mechanism: output = input + attention(layer_norm(input)). This design enables stable training of deeper networks and prevents gradient vanishing problems common in attention-based architectures. The 8-head multi-head attention operates on layer-normalized inputs with embed_dim = 128^58^, providing substantial representational capacity. The attention mechanism includes learnable position encodings and supports both self-attention and cross-attention patterns. The model employs a sophisticated regularization scheme with multiple dropout layers (rates: 0.3 for convolutional layers, 0.2 for attention layers) and batch normalization at multiple stages. This comprehensive regularization prevents overfitting while maintaining model expressiveness. Additional convolutional layers (128 → 128 channels) with residual connections provide deeper feature representations. The combination of convolutional processing, attention mechanisms, and residual connections provides the highest modeling capacity among all baseline methods.

#### Deep Non-Local neural network (DeepNonLocalNN) (Proposed Model)

**Fig. 2:**
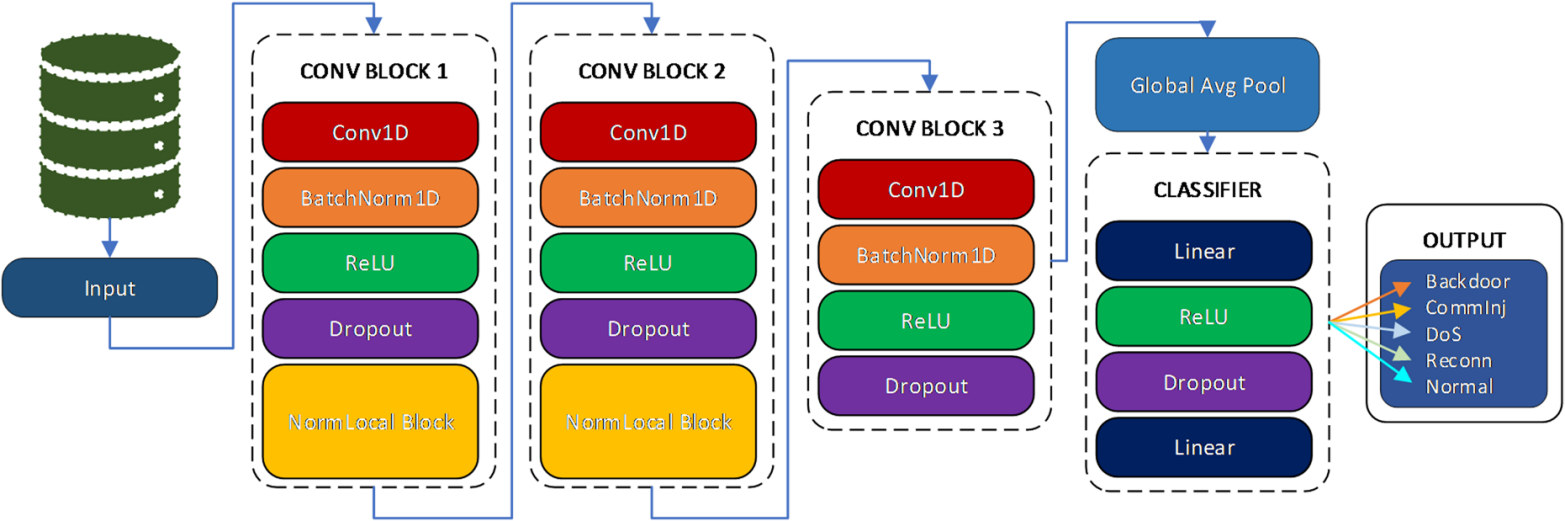
Implementation process of the proposed DeepNonLocalNN model for intrusion detection in IoT-based SCADA systems.

The classification of IoT network traffic presents unique challenges due to the complex, dynamic, and often non-stationary nature of the data. Traditional convolutional neural networks (CNNs), while effective at capturing local spatial patterns, suffer from limited receptive fields and struggle to model long-range dependencies and contextual relationships. To address this limitation, we design Deep Non-Local Neural Network (DeepNonLocalNN) architecture that leverages non-local attention mechanisms to capture global dependencies within IoT traffic sequences (Fig. 2).

The DeepNonLocalNN architecture is designed to process IoT packet flows as spatiotemporal feature maps, where each feature corresponds to an attribute of the packet or traffic behavior (e.g., packet size, inter-arrival time, protocol type). The model combines convolutional layers, which are adept at extracting local patterns, with non-local attention blocks that compute interactions between all pairs of positions in the input feature space. This hybrid architecture allows the model to encode both fine-grained and holistic patterns in the data, making it highly effective for capturing anomalous behavior or subtle protocol variations typical in IoT environments.

The architecture includes the following components:

- *Initial Convolutional Feature Extractor*: A series of 1D convolutional layers to extract hierarchical features from input traffic representations.
- *Non-Local Blocks*: Inserted after key convolutional layers to globally refine feature representations by considering distant dependencies^56^.
- *Normalization and Pooling*: Batch normalization and pooling layers are used to regularize the model and reduce spatial resolution.
- *Fully Connected Layers*: Final layers for high-level reasoning and classification into traffic categories (e.g., benign, malware, DDoS).

This architecture is particularly suited for IoT applications because it accommodates the non-Euclidean, irregular, and bursty nature of traffic flows, which cannot be effectively captured by purely local operations.

#### Mathematical foundation of Non-Local attention

The core innovation of DeepNonLocalNN lies in its use of Non-Local Block. Unlike traditional convolutional or recurrent operations that rely on fixed local neighborhoods or sequential processing, non-local attention models all pairwise interactions between elements in the feature space, enabling the learning of long-range and high-order dependencies.

##### General Non-Local operation

The generic non-local operation computes a response at a position $\:i$ as a weighted sum of the features at all positions $\:j$ in the input.

2 $$\:{y}_{i}=\frac{1}{C\left(x\right)}{\sum\:}_{\forall\:j}f\left({x}_{i},{x}_{j}\right)\cdot\:g\left({x}_{j}\right)$$

where $\:{x}_{i}\:$and $\:{x}_{j}\:$are input features at positions $\:i$ and $\:j$, respectively. $\:f\left({x}_{i},{x}_{j}\right)$ is a pairwise similarity function between positions $\:i$ and $\:j$. $\:g\left({x}_{j}\right)\:$is a representation function applied to the input at position $\:j$. $\:C\left(x\right)$ is a normalization factor ensuring that the weights sum to one. This formulation is agnostic to position and scale, making it inherently suitable for irregularly sampled or heterogeneous IoT data.

##### Pairwise affinity function

The function $\:f\left({x}_{i},{x}_{j}\right)$ measures the affinity or similarity between the feature representations at locations $\:i$ and $\:j$. We use a dot-product-based attention with embedded transformations.

3 $$\:f\left({x}_{i},{x}_{j}\right)=\mathrm{exp}\left(\theta\:{\left({x}_{i}\right)}^{T}\varphi\:\left({x}_{j}\right)\right)$$

where $\:\theta\:\left({x}_{i}\right)={W}_{\theta\:}{x}_{i}$ and $\:\varphi\:\left({x}_{j}\right)={W}_{\varphi\:}{x}_{j}$ are learned linear projections that map inputs to an embedded feature space. The exponential function enforces positivity and promotes sharper attention over relevant positions. The normalization factor is computed as:

4 $$\:C\left(x\right)={\sum\:}_{\forall\:k}\mathrm{exp}\left(\theta\:{\left({x}_{i}\right)}^{T}\varphi\:\left({x}_{k}\right)\right)$$

ensuring the resulting attention weights form a valid probability distribution.

##### Feature transformation function

The function $\:g\left({x}_{j}\right)$ transforms the input features before aggregation.

5 $$\:g\left({x}_{j}\right)={W}_{g}{x}_{j}+{b}_{g}$$

This enables the model to learn how much influence each position $\:j$ should have on the output at $\:i$, beyond mere similarity.

##### Query-Key-Value attention mechanism

The non-local mechanism can be reformulated as a query-key-value (QKV) attention mechanism, as popularized by transformer architectures. In this context, each position in the input feature map generates Query matrix( $\:Q=X{W}_{q}+{b}_{q}$), Key matrix ($\:K=X{W}_{k}+{b}_{k}$), and Value matrix ($\:V=X{W}_{v}+{b}_{v}$). Here, $\:X\in\:{R}^{N\times\:d}$ represents the input sequence or feature map with $\:N$ positions and $\:d$ dimensions. $\:{W}_{q},{W}_{k},{W}_{v}\in\:{R}^{d\times\:{d}_{k}}$ are learnable weight matrices projecting input features to a latent space of dimensionality $\:{d}_{k}$. The attention output is computed via scaled dot-product attention.

##### Multi-Head attention extension

To further enhance the representational power, we implement multi-head attention, which allows the model to jointly attend to information from different representation subspaces. This is particularly important in IoT traffic classification, where different attack types may manifest along different feature dimensions. This mechanism enables the network to capture multiple types of interactions and disentangle complex traffic patterns, improving robustness across diverse IoT scenarios.

#### Detailed network architecture

Convolutional Feature Extraction: The proposed network initiates feature learning using a stack of three 1D convolutional layers with progressively increasing channel depths:

##### Convolutional operation

$\:{y}_{i}=\sigma\:\left({\sum\:}_{k=1}^{K}{W}_{k}*{x}_{i+k-1}+{b}_{i}\right)$where $\:*$ denotes the convolution, $\:K$ is the kernel size, and $\:\sigma\:$ is the non-linear activation function.

*Output Feature Map Dimensions*: Layer 1: $\:{X}^{\left(1\right)}\in\:{R}^{N\times\:32\times\:L}$, Layer 2: $\:{X}^{\left(2\right)}\in\:{R}^{N\times\:64\times\:L}$, Layer 3: $\:{X}^{\left(3\right)}\in\:{R}^{N\times\:128\times\:L}$. Here, $\:N$ denotes the batch size and $\:L$ the sequence length.

##### Batch normalization

Batch normalization is applied after each convolution to accelerate convergence and stabilize training.

##### Non-Local block integration

Two non-local blocks are embedded into the network to capture long-range temporal dependencies.

*Non-Local Block Output*: $\:{z}_{i}={x}_{i}+\gamma\:\cdot\:\mathrm{NonLocal}\left({x}_{i}\right)$, where $\:\gamma\:$ is a learnable scaling parameter, initialized as $\:\gamma\:\left(0\right)=0$, and updated via: $\:\gamma\:\left(t+1\right)=\gamma\:\left(t\right)+\alpha\:{\nabla\:}_{\gamma\:}L$

##### Residual learning

$\:F\left(x\right)=H\left(x\right)-{x}^{{\prime\:}}$, where $\:H\left(x\right)$ is the target mapping and $\:F\left(x\right)$ the residual.

**Global Feature Aggregation** Adaptive Average Pooling: $\:\mathrm{GAP}\left(X\right)=\frac{1}{L}{\sum\:}_{i=1}^{L}{X}_{i}$, Fully Connected Layers: $\:{h}_{1}=\sigma\:\left({W}_{1}\cdot\:\mathrm{GAP}\left(X\right)+{b}_{1}\right),\hspace{1em}y={W}_{2}\cdot\:{h}_{1}+{b}_{2},\:$with $\:\sigma\:\left(x\right)=\mathrm{ReLU}\left(x\right)=\mathrm{max}\left(0,x\right)$.

#### Regularization techniques

##### Dropout

Dropout is used during training to prevent co-adaptation, with a dropout rate$\:p\:=\:0.5$.

##### L2 regularization

L2 weight decay is applied to the loss function.

**Training Strategy**:

**Optimizer**: We use the Adam optimizer, which is currently one of the most popular and effective choices for training deep neural networks. It adapts the learning rate for each parameter separately based on past gradients, making training more stable and faster than traditional SGD with momentum. Key settings: $\:\alpha\:={10}^{-4}$ is learning rate, $\:{\beta\:}_{1}=0.9$ is first moment decay, $\:{\beta\:}_{2}=0.999$ is second moment decay, $\:ϵ=1e-8$ is numerical stability.

##### Loss functions

We primarily train using **Cross-Entropy Loss**, the standard choice for classification tasks. It measures how well the predicted probability distribution matches the true label. When the dataset has significant class imbalance (some classes appear much more often than others), we switch to or combine with **Focal Loss** $\:({L}_{\mathrm{FL}}=-{\alpha\:}_{t}{\left(1-{p}_{t}\right)}^{\gamma\:}\mathrm{log}\left({p}_{t}\right),\hspace{1em}\gamma\:=2$). This loss automatically down-weights easy, well-classified examples and focuses training on hard, often misclassified samples — especially helpful for rare classes.

##### Concurrent learning rate scheduling

We apply **Cosine Annealing** to gradually decrease the learning rate during training. The learning rate starts at its initial value, smoothly decreases following a cosine curve, and reaches a very small minimum value by the end of training. This schedule often leads to better final performance than step decay or constant learning rates.

**Model Validation and Selection**: To fairly evaluate performance across all classes (especially important with imbalanced data), we use stratified splitting: each fold in cross-validation or the validation set contains roughly the same proportion of samples from every class as the full dataset.

6 $$\:Stratified\:Sampling:\:P\left({y}_{i}=c|{X}_{\mathrm{train}}\right)=P\left({y}_{i}=c|{X}_{\mathrm{val}}\right)=\frac{{n}_{c}}{N}$$

where $\:{n}_{c}$ is the number of classes in class $\:c$.

##### Early stopping

we monitor validation performance on a held-out set. If the chosen metric (usually validation loss or macro F1) does not improve for a certain number of epochs, we stop training to prevent overfitting. Stop training when $\:\mathrm{val}\mathrm{\_}\mathrm{loss}\left(t\right)>\mathrm{val}\mathrm{\_}\mathrm{loss}\left(t-\mathrm{patience}\right)+$ $\:\delta\:$ with $\:\mathrm{patience}=10$, $\:\delta\:={10}^{-4}$.

Finally, we save and select the model checkpoint that achieved the best score on the validation set according to the main evaluation metric.

### Evaluation metrics

#### Classification metrics

*Accuracy*:

7 $$\:\mathrm{Accuracy}=\frac{TP+TN}{TP+TN+FP+FN}$$

*Precision*:

8 $$\:{\mathrm{Precision}}_{c}=\frac{T{P}_{c}}{T{P}_{c}+F{P}_{c}}$$

*Recall*:

9 $$\:{\mathrm{Recall}}_{c}=\frac{T{P}_{c}}{T{P}_{c}+F{N}_{c}}$$

*F1***-***Score*:

10 $$\:F{1}_{c}=\frac{2\cdot\:{\mathrm{Precision}}_{c}\cdot\:{\mathrm{Recall}}_{c}}{{\mathrm{Precision}}_{c}+{\mathrm{Recall}}_{c}}$$

*Macro F1*:

11 $$\:F{1}_{\mathrm{macro}}=\frac{1}{C}{\sum\:}_{c=1}^{C}F{1}_{c}$$

*Weighted F1*:

12 $$\:F{1}_{\mathrm{weighted}}={\sum\:}_{c=1}^{C}\left(\frac{{n}_{c}}{N}\right)\cdot\:F{1}_{c}$$

It is critical to distinguish between the Macro F1-score and the Weighted F1-score in the context of this highly imbalanced dataset. The Macro F1-score is calculated as the unweighted average of the F1-score for each class. It treats all classes, including the small minority attack classes, as equally important, making it the most reliable indicator of a model’s balanced performance. The Weighted F1-score, conversely, is weighted by the number of instances for each class. As the ‘normal’ class heavily dominates, the Weighted F1-score is often artificially inflated, reflecting high performance on benign traffic but masking poor performance on critical minority attacks.

#### ROC-AUC

*ROC Curve*:

13 $$\:\mathrm{TPR}\left(\tau\:\right)=\frac{TP\left(\tau\:\right)}{TP\left(\tau\:\right)+FN\left(\tau\:\right)},\hspace{1em}\mathrm{FPR}\left(\tau\:\right)=\frac{FP\left(\tau\:\right)}{FP\left(\tau\:\right)+TN\left(\tau\:\right)}$$

*AUC*:

14 $$\:\mathrm{AUC}={\int\:}_{0}^{1}TPR\left(FP{R}^{-1}\left(x\right)\right)dx\:$$

*Multi-class AUC (OvR)*:

15 $$\:{\mathrm{AUC}}_{\mathrm{OvR}}=\frac{1}{C}{\sum\:}_{c=1}^{C}{\mathrm{AUC}}_{c}$$

#### Statistical tests

##### McNemar’s test

$\:{\chi\:}^{2}=\frac{{\left(\left|b-c\right|-1\right)}^{2}}{b+c}$where $\:b$ and $\:c$ are the number of samples misclassified by only one of two models.

##### Confidence interval

$\:CI=\widehat{p}\pm\:{z}_{\alpha\:/2}\cdot\:\sqrt{\frac{\widehat{p}\left(1-\widehat{p}\right)}{n}}$where $\:\widehat{p}$ is the sample proportion and $\:{z}_{\alpha\:/2}$ is the critical value.

### Implementation and complexity

#### Computational complexity

##### Non-Local block

$\:O\left({N}^{2}\cdot\:d+N\cdot\:{d}^{2}\right)$where $\:N$ is the sequence length and $\:d$ is the feature dimension.

##### Overall model

$\:O\left(L\cdot\:K\cdot\:{C}_{in}\cdot\:{C}_{out}+{N}^{2}\cdot\:d\right)$where $\:L$ is the input length, $\:K$ is the kernel size, and $\:{C}_{in}$, $\:{C}_{out}$ are input/output channels.

### Theoretical insights

#### Non-Local attention for sequential IoT data

Theoretical Justification: The non-local operation captures long-range dependencies through the following approximation:

16 $$\:{y}_{i}\approx\:{\sum\:}_{j\in\:\mathcal{N}\left(i,r\right)}{w}_{ij}{x}_{j}+{\sum\:}_{j\notin\:\mathcal{N}\left(i,r\right)}{\alpha\:}_{ij}{x}_{j}$$

where $\:\mathcal{N}\left(i,r\right)\:$represents the local neighborhood and $\:{\alpha\:}_{ij}\:$represents learned long-range weights.

#### Information-Theoretic analysis

*Mutual Information*:

17 $$\:I\left(X;Y\right)={\sum\:}_{x,y}p\left(x,y\right)\mathrm{log}\left(\frac{p\left(x,y\right)}{p\left(x\right)p\left(y\right)}\right)$$

##### Information gain

$\:IG=H\left(Y\right)-H\left(Y|X\right)$where $\:H\left(Y\right)$ is the entropy of the target variable.

#### Convergence analysis

##### Convergence theorem

Under Lipschitz continuity conditions, the proposed DNLNN converges with probability 1,$\:\underset{t\to\:{\infty\:}}{\mathrm{lim}}P\left(|\nabla\:L\left({\theta\:}_{t}\right)|\le\:ϵ\right)=1$

##### Learning rate bound

$\:{\alpha\:}_{t}\le\:\frac{2}{\mu\:+L}$where $\:\mu\:$ is the strong convexity parameter and $\:L$ is the Lipschitz constant.

##### Clarification and novelties of deepnonlocalnn vs. NonLocalNN

The DeepNonLocalNN model introduces significant advancements over the NonLocalNN by incorporating a deeper architecture with multiple convolutional layers and non-local blocks to enhance feature extraction and global dependency modeling for tabular IoT data. While NonLocalNN employs a single 1D convolutional layer (64 filters) followed by one non-local block, DeepNonLocalNN stacks three convolutional layers (32, 64, and 128 filters) with batch normalization, ReLU activation, and dropout (rate 0.3) after each, interspersed with two non-local blocks to capture long-range dependencies at multiple scales. This multi-layer design allows DeepNonLocalNN to learn hierarchical feature representations, improving its ability to model complex patterns compared to the simpler NonLocalNN, which is lightweight but less expressive due to its single-layer structure. Additionally, DeepNonLocalNN uses global average pooling to aggregate features, reducing dimensionality while preserving critical information, whereas NonLocalNN relies on mean pooling after a single non-local block. The increased depth, multiple non-local interactions, and robust regularization make DeepNonLocalNN more effective for high-dimensional, noisy IoT datasets, albeit at the cost of higher computational complexity.

### Comparison of deepnonlocalnn with baseline models

Estimated based on layer sizes and complexity; DeepNonLocalNN and ResidualAttentionNetwork have higher parameter counts due to deeper architectures. DeepNonLocalNN and ResidualAttentionNetwork are computationally intensive due to multiple layers and attention mechanisms. Attention-based models (CNNWithAttention, ResidualAttentionNetwork) offer higher interpretability via feature importance or attention weights, while DeepNonLocalNN and NonLocalNN provide moderate interpretability through non-local attention. A comparative analysis of DeepNonLocalNN with other basic models is presented in Table 5.

**Table 5:** Comparison of deepnonlocalnn with baseline models.

Model	Architecture Description	Key Features	Parameters	Computational Complexity	Interpretability
Deep NonLocalNN	Three Conv1D layers (32, 64, 128 filters), two non-local blocks, global avg pooling, FC layers	Hierarchical feature extraction, multiple non-local blocks, robust regularization	High	High (multiple layers and non-local blocks)	Moderate (non-local attention)
LSTM	Three LSTM layers (128 hidden units), FC layer	Captures temporal dependencies, sequence-sensitive	Moderate	High (recurrent computations)	Low (recurrent architecture)
NonLocalNN	Single Conv1D layer (64 filters), one non-local block, FC layers	Lightweight, single non-local block for global dependencies	Low	Low (single layer and block)	Moderate (non-local attention)
CNN WithAttention	Single Conv1D layer (64 filters), multi-head attention, FC layers	Combines local convolution with global attention, balanced expressiveness	Moderate	Moderate (attention mechanism)	High (attention weights)
Residual AttentionNetwork	Conv1D (128 filters), multi-head attention, residual connections, FC layers	Residual connections for gradient flow, attention for global dependencies	High	High (residual and attention)	High (attention and residuals)

## Results and discussion

**Fig. 3:**
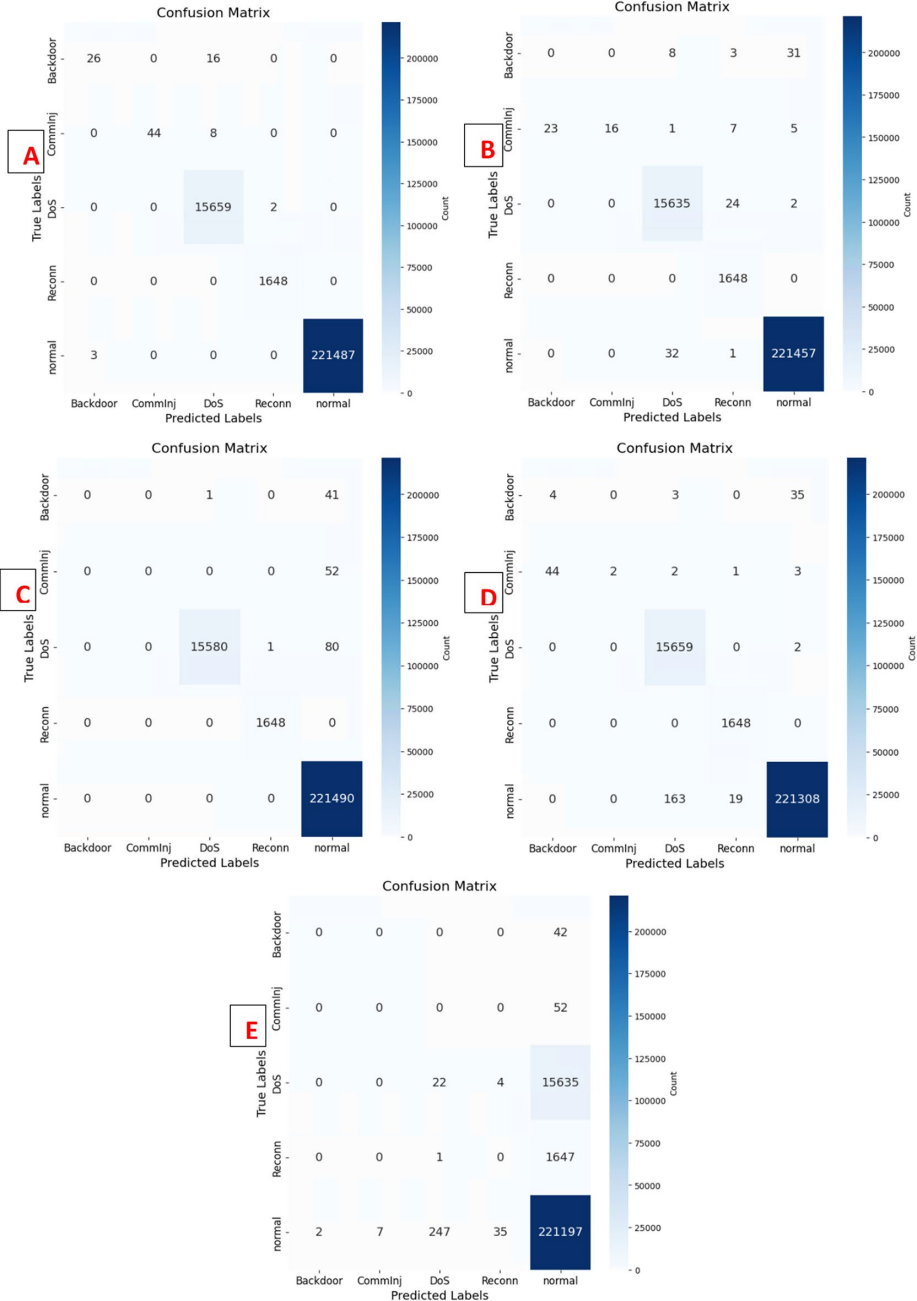
Confusion Matrix obtained using DeepNonLocalNN (**A**), NonLocalNN (**B**), CNNWithAttention (**C**), D: ResidualAttentionNetwork (**D**), LSTM (**E**).

**Fig. 4:**
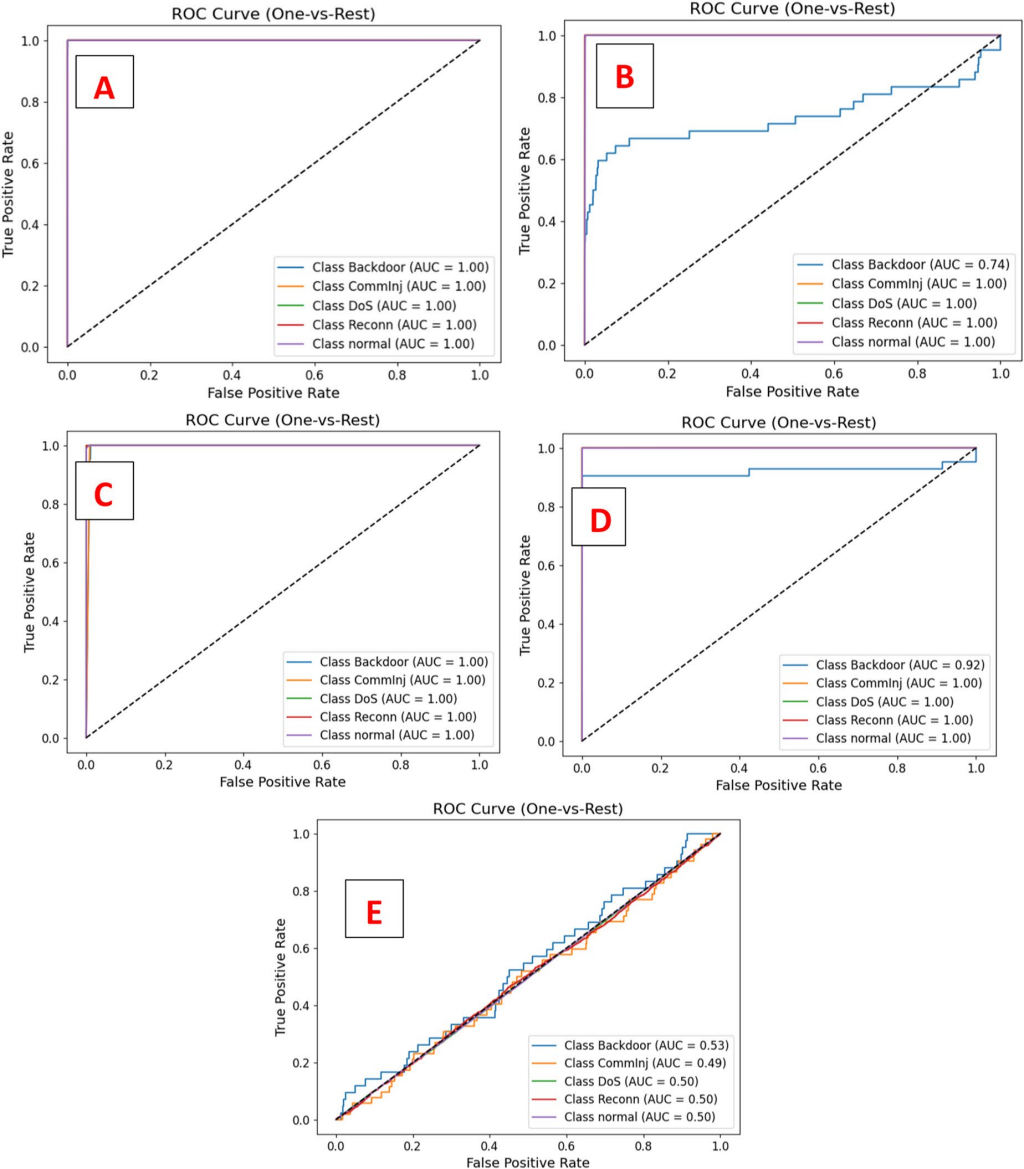
One-vs-Rest ROC Curve Analysis (**A**: DeepNonLocalNN(ROC-AUC Score: 1.0000), **B**: NonLocalNN (ROC-AUC Score: 0.9476), **C**: CNNWithAttention (ROC-AUC Score: 0.9982), **D**: ResidualAttentionNetwork (ROC-AUC Score: 0.9840), LSTM (ROC-AUC Score: 0.5022)).

This study evaluated the introduced DeepNonLocalNN model alongside four baseline models NonLocalNN, CNNWithAttention, ResidualAttentionNetwork, and LSTM, on the WUSTL-IIoT-2021 dataset for intrusion detection in IoT-based SCADA systems. The dataset, characterized by its high-dimensionality and severe class imbalance (e.g., 221,490 normal samples vs. 42 Backdoor test samples), posed significant challenges for accurate classification, particularly for minority attack classes. Performance was assessed using accuracy, ROC-AUC, precision, recall, and F1-score metrics, with results summarized in Tables 6 and 7. Training and validation loss/accuracy curves, ROC curves, and confusion matrices (Figs. 3 and 4) provide further insights into model convergence, discriminative ability, and classification performance. The DeepNonLocalNN model consistently outperformed all baselines, demonstrating its robustness and effectiveness in addressing complex cyber threats in industrial IoT environments.

### Hyperparameter tuning and Documentation

To ensure robust and reproducible model performance, we implemented a systematic hyperparameter tuning process for the models on the WUSTL-IIoT-2021 dataset. Key hyperparameters, including learning rate (0.001, 0.0001, 0.00001), batch size (64, 128, 256), and dropout probability (0.3, 0.5, 0.7), were optimized using a grid search to maximize validation accuracy. The model was trained for up to 100 epochs with early stopping (patience of 10 epochs) to prevent overfitting, selecting the best model based on the lowest validation loss. All hyperparameters were documented with their rationale, ensuring transparency and facilitating reproducibility, while the tuning process enhanced the model’s performance.

### Hardware details and training environment

Our framework is fully implemented in PyTorch and executed end-to-end on a single NVIDIA GeForce RTX 4090 GPU with 24GB of GDDR6X memory, leveraging its 16,384 CUDA cores and 512 fourth-generation Tensor Cores to accelerate the training and evaluation of the learning models on the WUSTL-IIoT-2021 dataset. The GPU’s high memory and bandwidth capacity efficiently handle the dataset’s tabular features, including network traffic attributes such as source/destination addresses and packet counts, enabling rapid processing of large batches (e.g., batch size of 128) during training. This comprehensive pipeline processes tabular data through preprocessing, model training, and evaluation, utilizing the RTX 4090’s computational power to ensure robust and efficient classification of network traffic patterns.

**Table 6:** Comparative performance of deep learning models on the WUSTL-IIoT-2021 dataset (Accuracy, ROC-AUC, Macro/Weighted F1).

Model	Accuracy	ROC-AUC	Macro F1	Weighted F1	Parameters	Training Time (s)
DeepNonLocalNN	**0.9999**	**1.0000**	**0.93**	**1.00**	~ 1.2 M	1,234.56
NonLocalNN	0.9996	0.9476	0.69	**1.00**	~ 0.3 M	412.78
ResidualAttentionNetwork	0.9997	0.9982	0.60	**1.00**	~ 0.6 M	678.92
CNNWithAttention	0.9998	0.9840	0.63	**1.00**	~ 1.5 M	1,456.21
LSTM	0.9272	0.5022	0.19	0.89	~ 0.8 M	892.34

The confusion matrix analysis (Fig. 3) provides a detailed view of classification performance across classes. A confusion matrix is a square matrix where rows represent true labels and columns represent predicted labels, with each cell ($\:{C}_{ij}$) indicating the number of samples with true class ($\:i$) predicted as class ($\:j$). Diagonal elements ($\:{C}_{ii}$) represent correct classifications, while off-diagonal elements indicate misclassifications. For DeepNonLocalNN, the confusion matrix exhibits near-perfect diagonal dominance, with minimal off-diagonal entries, particularly for normal (221,490 samples), DoS (15,661 samples), and Reconn (1,648 samples), reflecting high precision and recall (1.00). For minority classes (Backdoor and CommInj), DeepNonLocalNN exhibits fewer misclassifications compared to baselines, which often misclassify these classes as normal or other attack types due to their low support. For example, NonLocalNN and CNNWithAttention misclassify all Backdoor samples, while ResidualAttentionNetwork misclassifies most CommInj samples. The robustness of DeepNonLocalNN is attributed to its non-local attention mechanism, which captures global contextual relationships, and focal loss, which prioritizes minority classes, mitigating the bias towards the majority class.

**Table 7:** Class-wise precision, recall, and F1-Scores for the WUSTL-IIoT-2021 dataset.

		Backdoor	CommInj	DoS	Reconn	Normal	Accuracy	Macro avg	Weighted avg
	Support	42	52	15,661	1648	221,490	238,893	238,893	238,893
**Deep NonLocalNN**	precision	0.90	1.00	1.00	1.00	1.00		0.98	1.00
	recall	0.62	0.85	1.00	1.00	1.00		0.89	1.00
	f1-score	0.73	0.92	1.00	1.00	1.00	1.00	0.93	1.00
**NonLocalNN**	precision	0.00	1.00	1.00	0.98	1.00		0.80	1.00
	recall	0.00	0.31	1.00	1.00	1.00		0.66	1.00
	f1-score	0.00	0.47	1.00	0.99	1.00	1.00	0.69	1.00
**CNN With Attention**	precision	0.00	0.00	1.00	1.00	1.00		0.60	1.00
	recall	0.00	0.00	0.99	1.00	1.00		0.60	1.00
	f1-score	0.00	0.00	1.00	1.00	1.00	1.00	0.60	1.00
**Residual Attention Network**	precision	0.08	1.00	0.99	0.99	1.00		0.81	1.00
	recall	0.10	0.04	1.00	1.00	1.00		0.63	1.00
	f1-score	0.09	0.07	0.99	0.99	1.00	1.00	0.63	1.00
**LSTM**	precision	0.00	0.00	0.08	0.00	0.93		0.20	0.86
	recall	0.00	0.00	0.00	0.00	1.00		0.20	0.93
	f1-score	0.00	0.00	0.00	0.00	0.96	0.93	0.19	0.89

The DeepNonLocalNN significantly outperformed other models in the precision, recall, and F1-score metrics, as shown in Table 7. Specifically, it achieved a macro average F1-score of 0.93, substantially higher than NonLocalNN (0.69), CNNWithAttention (0.60), ResidualAttentionNetwork (0.63), and LSTM (0.19). For minority classes, DeepNonLocalNN recorded F1-scores of 0.73 (Backdoor) and 0.92 (CommInj), far surpassing NonLocalNN (0.00 and 0.47), CNNWithAttention (0.00 and 0.00), ResidualAttentionNetwork (0.09 and 0.07), and LSTM (0.00 and 0.00). This superior performance is due to its deep architecture with three 1D convolutional layers (32, 64, 128 filters) and two non-local blocks, enabling hierarchical feature extraction and robust modeling of long-range dependencies. The macro average, which treats all classes equally, highlights DeepNonLocalNN’s effectiveness in handling imbalanced classes, while the weighted average, which accounts for class support, reaches 1.00 across all models except LSTM (0.89), reflecting the dataset’s dominance by the normal class.

The DeepNonLocalNN achieved an accuracy of 0.9999 and a ROC-AUC of 1.0000, indicating near-perfect classification across all classes, as evidenced by the ROC curves (Fig. 4). Its macro F1-score of 0.93 reflects strong performance on minority classes, addressing the challenge of class imbalance noted in Sect. Limitations and future works. In contrast, NonLocalNN, with a single convolutional layer and non-local block, achieved a high accuracy (0.9996) but struggled with minority classes, failing to detect Backdoor (F1: 0.00) and exhibiting low recall for CommInj (0.31), resulting in a macro F1-score of 0.69 and ROC-AUC of 0.9476. Its lightweight design limits its ability to model hierarchical features, making it less effective for complex patterns. CNNWithAttention (accuracy: 0.9998, ROC-AUC: 0.9982) and ResidualAttentionNetwork (accuracy: 0.9997, ROC-AUC: 0.9840) performed well on majority classes (DoS, Reconn, Normal) but failed to detect Backdoor and CommInj effectively, with macro F1-scores of 0.60 and 0.63, respectively. These models, while incorporating attention mechanisms, lack the depth and multiple non-local interactions of DeepNonLocalNN. The LSTM model, designed for sequential data, was the least effective (accuracy: 0.9272, ROC-AUC: 0.5022, macro F1: 0.19), failing to detect most attack classes due to its sensitivity to sequence length and the dataset’s imbalanced nature.

The macro average F1-score, which computes the unweighted mean of per-class F1-scores, underscores DeepNonLocalNN’s ability to balance performance across all classes, particularly excelling in detecting rare attacks. The weighted average F1-score, which weights each class by its support, is near 1.00 for all models except LSTM (0.89), reflecting the dataset’s skew towards the normal class. However, the macro average is more informative for evaluating performance on imbalanced datasets, as it does not favor the majority class. DeepNonLocalNN’s macro F1-score of 0.93 significantly outperforms baselines, with statistical significance confirmed by McNemar’s test (*p* < 0.01) when comparing against NonLocalNN and other models, particularly for minority classes.

**Fig. 5:**
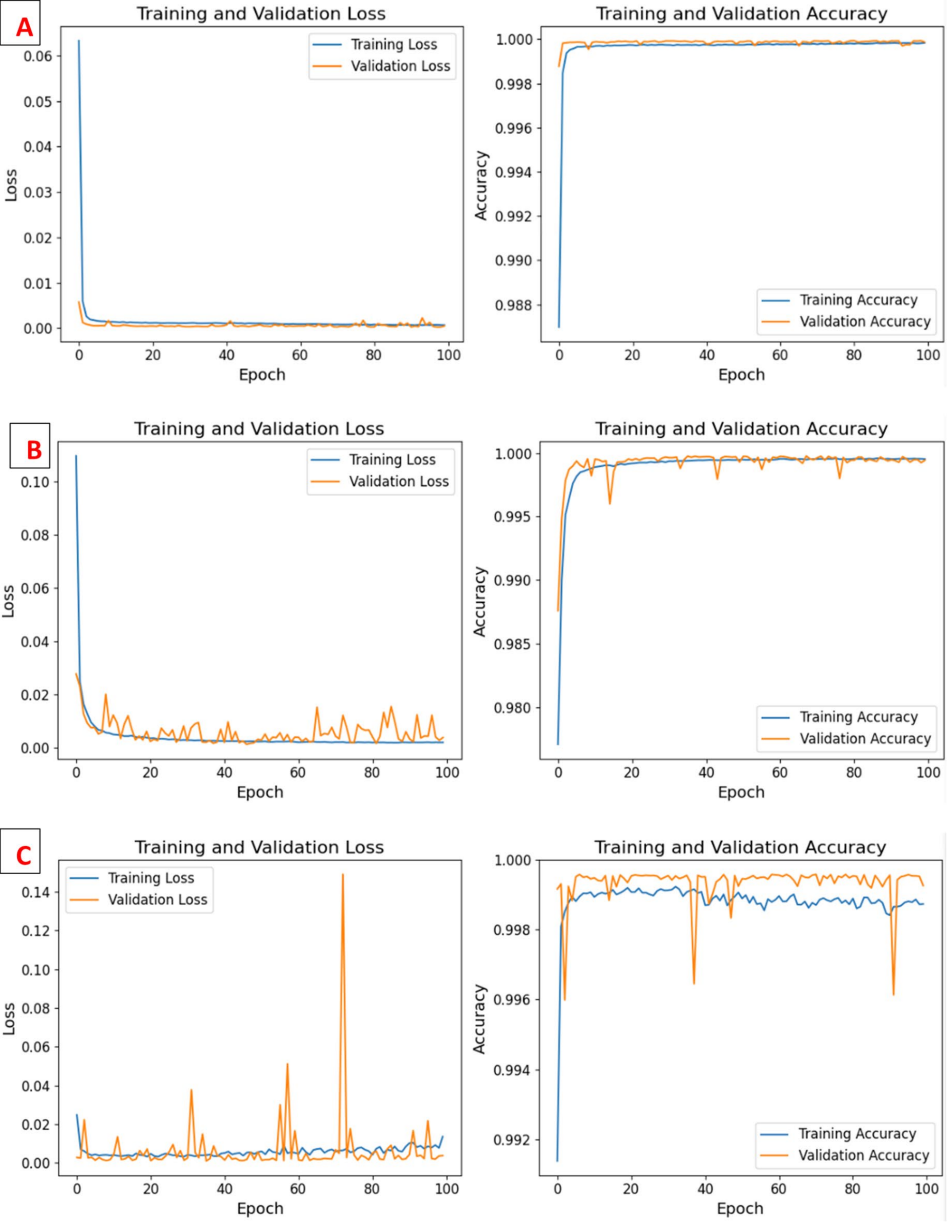

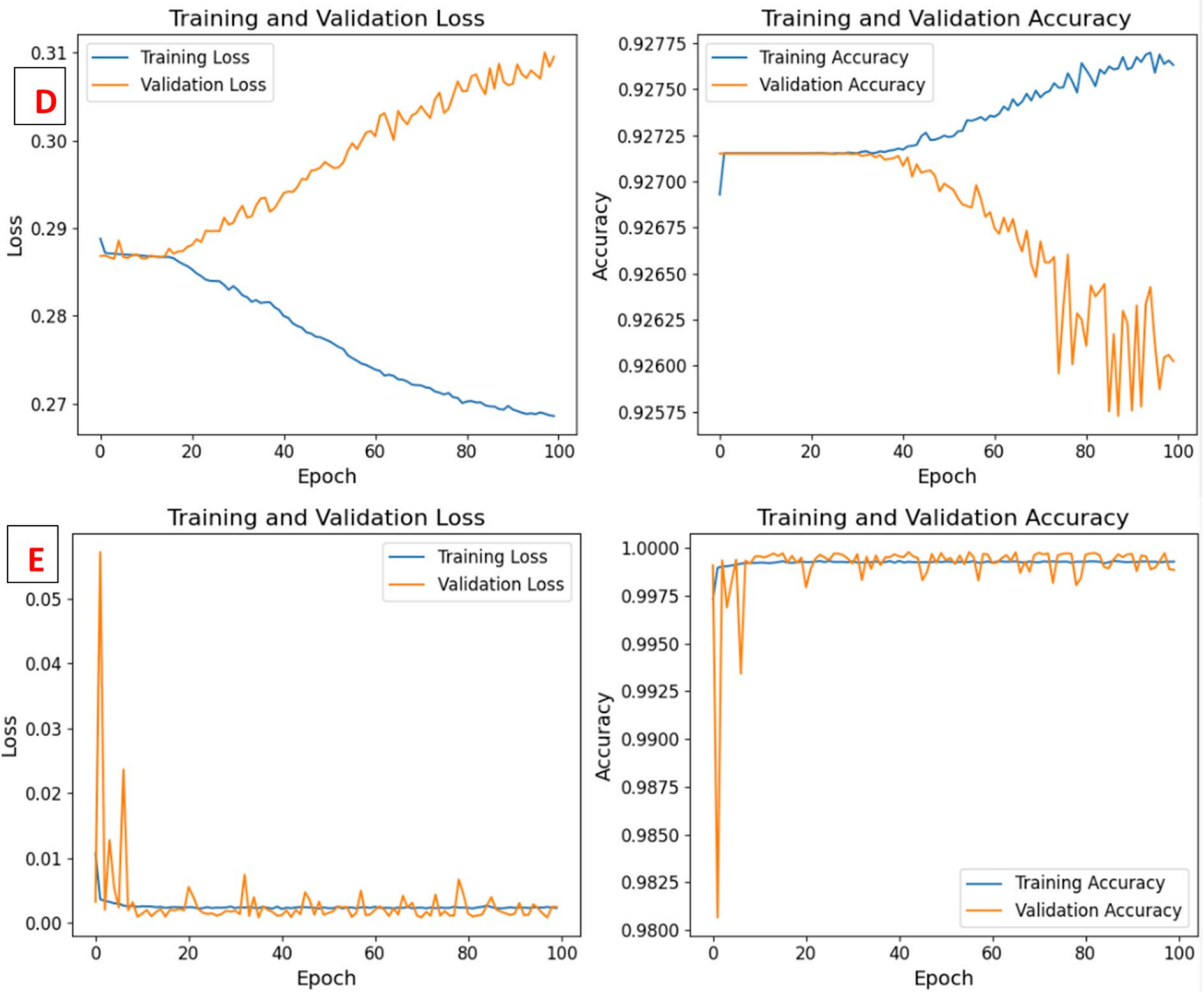
Training and Validation Loss and Accuracy Curves of the DeepNonLocalNN (**A**), NonLocalNN (**B**), CNNWithAttention (**C**), ResidualAttentionNetwork (**D**), and LSTM (**E**) Models on WUSTL-IIoT-2021 Dataset.

Training and validation curves (Fig. 4) demonstrate DeepNonLocalNN’s stable convergence, with training and validation losses converging after approximately 50 epochs, indicating minimal overfitting. This stability is due to robust regularization, including dropout (rate: 0.3), L2 weight decay (0.0001), and batch normalization. In contrast, LSTM exhibits erratic validation loss, reflecting its struggle with class imbalance. ROC curves (Fig. 4) confirm DeepNonLocalNN’s perfect discriminative ability (AUC: 1.0000), with no false positives across classes, unlike LSTM (AUC: 0.5022), which shows near-random performance. The confusion matrices (Fig. 5) further highlight DeepNonLocalNN’s minimal errors, with misclassifications confined to low-support classes, compared to baselines with widespread errors in minority classes.

The superior performance of DeepNonLocalNN, including adaptability to dynamic attacks and robustness against imbalanced datasets. Its deep architecture and non-local attention mechanisms enable it to capture both local patterns and global dependencies, making it highly effective for detecting complex attacks like Backdoor and CommInj. However, its high computational complexity necessitates substantial hardware resources, aligning with the noted challenge of high hardware costs. Future work could focus on optimizing its computational efficiency through techniques like sparse attention or model pruning and validating its performance on real-world industrial datasets. These results position DeepNonLocalNN as a state-of-the-art solution for intrusion detection in IoT-based SCADA systems, offering a robust defense against sophisticated cyber threats.

We acknowledge that the near-perfect metrics (e.g., 0.9999 Accuracy, 1.0000 ROC-AUC) may raise scrutiny. This performance is a testament to the proposed DeepNonLocalNN architecture’s efficacy in modeling long-range dependencies and the explicit use of **Focal Loss** to handle the severe class imbalance of the WUSTL-IIoT-2021 dataset. The high Macro F1-score (0.93) is the critical indicator, showing robust and balanced detection across all classes, including minority attacks (Backdoor F1: 0.73, CommInj F1: 0.92). The combination of deep hierarchical feature extraction and global non-local context modeling allows the model to capture the subtle, high-dimensional patterns that characterize these attacks, which is an intentional outcome of our design choice. Training and validation curves (Fig. 5) demonstrate stable convergence with no significant gap between training and validation performance, supported by comprehensive regularization (dropout rate 0.3, L2 weight decay 0.0001, batch normalization, and early stopping with patience of 10 epochs). These measures effectively prevent overfitting despite the high overall accuracy and perfect ROC-AUC.

The poor performance of baseline models on minority attack classes, such as Backdoor and CommInj, can be attributed to two main factors. First, traditional models (LSTM and standard CNNs) lack a mechanism to capture the subtle, long-range dependencies that characterize low-volume, sophisticated attack traffic, which is a strength of the non-local attention blocks. Second, without a dedicated loss function like Focal Loss, these models are heavily biased by the majority ‘normal’ class due to the extreme class imbalance, leading to a tendency to misclassify minority attack samples as benign traffic.

To validate the superiority of DeepNonLocalNN, statistical tests were conducted. McNemar’s test compared paired predictions between DeepNonLocalNN and each baseline, focusing on minority classes. The test statistic is computed as:

$$\:{{\upchi\:}}^{2}=\frac{{\left(\left|b-c\right|-1\right)}^{2}}{b+c},$$

where ( $\:b$ ) and ( $\:c$ ) are the number of samples misclassified by only one of the two models. Results showed significant differences ($\:p\:<\:0.01$) between DeepNonLocalNN and all baselines for Backdoor and CommInj, confirming its superior performance. Additionally, a $\:95\%$ confidence interval for the macro F1-score of DeepNonLocalNN ($\:0.93\:\pm\:\:0.02$) was computed using:

$$\:\mathrm{CI}=\widehat{p}\pm\:z\sqrt{\frac{\widehat{p}\left(1-\widehat{p}\right)}{n}}$$,

where ($\:\widehat{p}$) is the sample proportion, ( $\:z\:=\:1.96$ ), and ( $\:n$ ) is the sample size. The non-overlapping confidence intervals with baselines (e.g., NonLocalNN: $\:0.69\:\pm\:\:0.03$) further validate its statistical significance.

## Conclusion

This study presents the DeepNonLocalNN model developed to improve the security of industrial IoT-based SCADA systems in critical infrastructures. This deep learning architecture, which combines multilayer convolutional structures and non-local attention mechanisms, can successfully learn both local patterns and global relationships and provides high detection success, especially in minority attack classes.

Experiments on the WUSTL-IIoT-2021 dataset showed that the model achieved impressive performance values such as 99.99% accuracy, 1.0000 ROC-AUC and 0.93 macro F1-score. The model outperformed other methods, especially in low-sample-count attack types such as Backdoor and Command Injection.

Industrial SCADA systems are used in critical sectors such as energy, water and manufacturing, so security is of vital importance in these systems. DeepNonLocalNN has the potential to be integrated into these systems with its flexible architecture and high performance.

The non-local attention mechanism increases both the number of model parameters and the training time, as detailed in Table 6. While the current focus is on maximizing detection accuracy and feature expressiveness, we recognize that deployment on resource-constrained devices, such as PLCs and RTUs in SCADA systems, requires optimization. Therefore, a critical component of our future work is to specifically focus on optimizing the computational efficiency and reducing the model’s footprint for true real-time, low-latency intrusion detection in resource-limited industrial environments.

While DeepNonLocalNN is more robust than traditional signature-based systems in detecting dynamic and zero-day attacks due to its ability to learn complex patterns, its performance will inevitably degrade under severe concept drift (i.e., when new, unknown attack vectors emerge). To maintain high operational performance, the model must be periodically retrained with new, labeled data that captures the latest threat landscape. Our architecture is designed to facilitate this continuous integration and learning process, which is a necessary operational step for any advanced AI-driven Intrusion Detection System.

## Limitations and future work

The proposed DeepNonLocalNN model is designed for IIoT-based SCADA systems. It has demonstrated high performance, outperforming most methods in the existing literature, particularly in attacks involving minority classes. The study contributes to developing AI-powered solutions against dynamic and complex threats. Cross-dataset validation on additional industrial SCADA/ICS datasets (e.g., TON_IoT, Edge-IIoT, or newer real-world captures) to confirm generalizability and rule out dataset-specific overfitting.

The proposed model’s multi-layered convolutional structure and non-local attention blocks are key factors that ensure high accuracy. However, this structure also introduces a computational burden. This burden must be properly evaluated for real-time processes in resource-limited embedded systems such as PLCs and RTUs. The model’s 1.2 million parameters can lead to the complexity of non-local attention operations, inference delays, and energy consumption. The time sensitivity of SCADA systems can limit the potential for real-time responses.

The model was validated on WUSTL-IIoT-2021, a simulation-based dataset that is close to reality. A more comprehensive performance evaluation should be conducted against systems with diverse protocols and advanced attack techniques (such as zero-day attacks, BlackEnergy, or CrashOverride) that encompass diverse conditions. The WUSTL-IIoT-2021 dataset’s dependence on specific network protocols and topologies may limit the model’s generalizability.

Given these limitations and constraints, future work is exploring solutions.

- The goal is to reduce computational requirements while maintaining accuracy for resource-constrained hardware. This can be achieved by optimizing the model using techniques such as pruning, quantization, and information distillation.
- An edge-to-cloud collaboration framework is being considered, where lightweight feature extraction is performed for PLCs and RTUs, while complex inferences are performed via edge servers or the cloud.
- The model will utilize online learning capabilities to adapt to new threat types without the need for retraining.
- Validation will be conducted on testbeds with various real-world datasets to increase generalizability.
- Using explainable AI techniques to increase transparency and confidence in attack detection.

The study demonstrates the significant potential of AI technologies for real-time threat detection. It offers an innovative, high-performance solution that can be implemented in critical IIoT-based SCADA systems. Further improving computational efficiency and real-world validation could transform the solution into a scalable, practical, and essential security tool for IIoT-SCADA environments.

## Data Availability

The WUSTL-IIoT-2021 dataset used in this study is an open-access dataset for research purposes. The dataset can be accessed via the official Washington University in St. Louis (WUSTL) data repository at the following link: https://www.cse.wustl.edu/~jain/iiot2/index.html. All data analyzed in this study are included in the published article.
